# The Forgotten Disease: A Case of Lemierre's Syndrome with Distal Extremity Involvement

**DOI:** 10.1155/2020/4346937

**Published:** 2020-03-16

**Authors:** John Gaskill, Michael Aronson

**Affiliations:** Lewis-Gale Hospital Montgomery, 3700 South Main St., Blacksburg, VA 24060, USA

## Abstract

Once coined the “Forgotten Disease,” Lemierre's syndrome is a rare condition that results from oropharyngeal infection with the gram-negative, anaerobic *Fusobacterium necrophorum*. The typical progression of illness involves spread to adjacent structures such as the internal jugular vein with resulting thrombophlebitis. Septic emboli to distant sites are also a common sequela. Here, we present a case of Lemierre's syndrome in a 20-year-old, otherwise healthy, male. The patient presented with fever, sore throat, and dysphagia. Imaging revealed peritonsillar multiloculated fluid collections and necrotizing pneumonia with multiple pulmonary abscesses. The patient's hospital course was complicated by the development of necrotizing fasciitis in his right lower leg, which required incision and drainage with surgical washout. In addition to systemic intravenous antibiotics and anticoagulation, he underwent multiple thoracentesis procedures. The patient was ultimately transferred to a tertiary care center due to persistent fevers and lung abscesses. This case highlights the challenges of initial diagnosis, as well as the treatment choices faced by the attending physicians.

## 1. Introduction

In 1936, the French bacteriologist Andre Lemierre published a report detailing the link between septicemia and pharyngitis that he had observed while working at the Claude Bernard Hospital in Paris [1]. The postanginal septicemia which now bears his name is most often precipitated by a suppurative oropharyngeal infection. The infection is typically caused by the gram-negative, anaerobic *Fusobacterium necrophorum*. Fusobacteria can be found as normal flora in the upper respiratory tract of humans [2]. Other anaerobic bacteria have been identified as sources of infection, but these are atypical [3]. The disease mostly affects young, otherwise healthy adults. The first symptom is typically a sore throat secondary to exudative tonsillitis or peritonsillar abscess. The infection then spreads into adjacent structures, such as the internal jugular vein and carotid artery. Thrombophlebitis of the neck veins can cause pain, swelling, and dysphagia. Sepsis with complicating metastatic spread of septic microemboli is the typical progression of illness [4]. The most common site of metastatic spread is the lungs, with 85% of cases demonstrating pulmonary infiltrates [5]. Other feared complications include spread of infection along the carotid sheath and into the mediastinum [4]. Mortality rate before advent of antibiotics was extremely high, with 18 of Andre Lemierre's 20 identified patients succumbing to their disease [1]. In the modern era of medicine, mortality rates range from 6.4 to 17% [6, 7].

## 2. Case Presentation

AT is a 20-year-old Caucasian male who was seen in the emergency department for evaluation of fever, chills, sore throat, and some difficulty swallowing. He was also complaining of mid-back pain, made worse with deep inspiration, and tender swollen glands in his neck. He denied any recent dental procedures or tooth infections. He was seen at his university health center for evaluation five days prior and was diagnosed with viral pharyngitis. He was prescribed a course of prednisone, which he had completed one day prior to presenting to the emergency department. His temperature in the emergency department was 106° Fahrenheit, and his heart rate was 150 beats per minute (bpm). He also complained of some pain in his right calf that he attributed to “slipping and hyperextending his ankle” several days prior. A computed tomography with contrast of his neck was obtained that showed an inflammatory and infectious process of the soft tissue posterior to the oral cavity and extending into the prevertebral soft tissue in the midline. The patient was then transferred to a nearby facility that had otorhinolaryngology coverage for further evaluation. He was admitted to the intensive care unit under sepsis protocol, where he continued to complain of a sore throat in addition to a cough productive of purulent sputum.

Physical examination at the time of admission revealed a swollen neck with fullness and tenderness in the anterior and posterior cervical lymph nodes bilaterally. He was unable to open his mouth more than 1.5–2 inches due to pain and swelling. His oropharynx appeared erythematous. He was breathing fast and shallow due to pain with deep inspiration, but he was still able to complete full sentences. He had decreased breath sounds at the bases bilaterally without wheezes or crackles. His right calf was moderately swollen compared to the left and was tender. He had good pulses in both dorsalis pedis and posterior tibialis in both legs in addition to full range of motion in his ankles. Initial lab results showed an elevated white count of 14.1 with 19% bands. His hemoglobin was 15.4, and platelet count was low at 46,000. Creatinine was 1.33, glucose 105, albumin low at 2.6, potassium 3.7, and calcium 8.5. Other lab values of note included alkaline phosphatase 151, aspartate aminotransferase (AST) 46, alanine aminotransferase (ALT) 30, lactate 2.1, and bilirubin 2.6.

**Figure 1:**
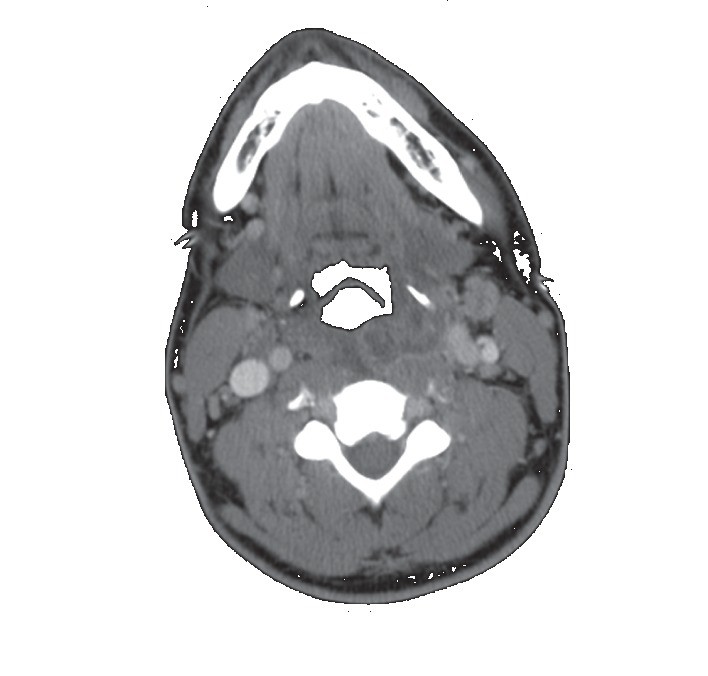
Horizontal section of neck computed tomography (CT) with contrast showing retropharyngeal and bilateral peritonsillar multiloculated enhancing fluid collections in addition to thrombosis of the left retromandibular vein and punctate foci within the left internal jugular vein.

**Figure 2:**
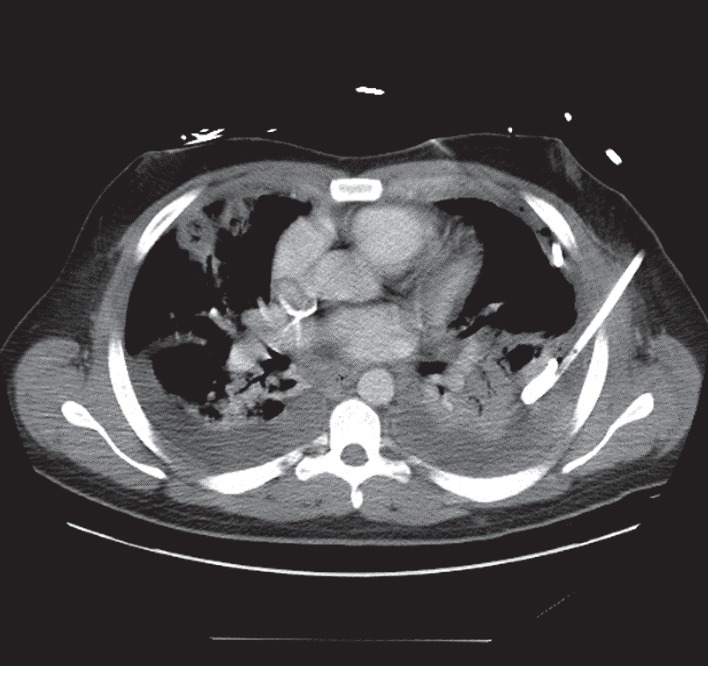
Horizontal section of chest computed tomography (CT) with contrast showing left-sided, multiloculated pleural effusion, status postplacement of pigtail catheter. Multifocal areas of necrotizing pneumonia and multiple pulmonary abscesses with smaller right-sided pleural effusion are also shown.

The treatment plan involved broad-spectrum coverage with administration of intravenous vancomycin and piperacillin/tazobactam, in addition to aggressive IV fluid repletion. The patient was started on low-molecular-weight heparin 40 mg subcutaneous daily for deep vein thrombosis prophylaxis. The chest computed tomography (CT) with contrast showed multifocal areas of necrotizing pneumonia with multiple pulmonary abscesses. A multiloculated effusion was also identified on the left side with a smaller free flowing effusion on the right side. A repeat neck computed tomography (CT) with contrast (Figure 1) showed persistent retropharyngeal and bilateral peritonsillar multiloculated enhancing fluid collections. It also showed thrombosis of the left retromandibular vein and punctate foci within the left internal jugular vein, which was thought to represent partial thrombosis. On the day of admission, the patient underwent computed tomography (CT) guided empyema drainage with placement of left-sided chest tube (Figure 2). The following day, a second chest tube was placed on the left side and a right-sided chest tube was placed on the third day of hospitalization.

**Figure 3:**
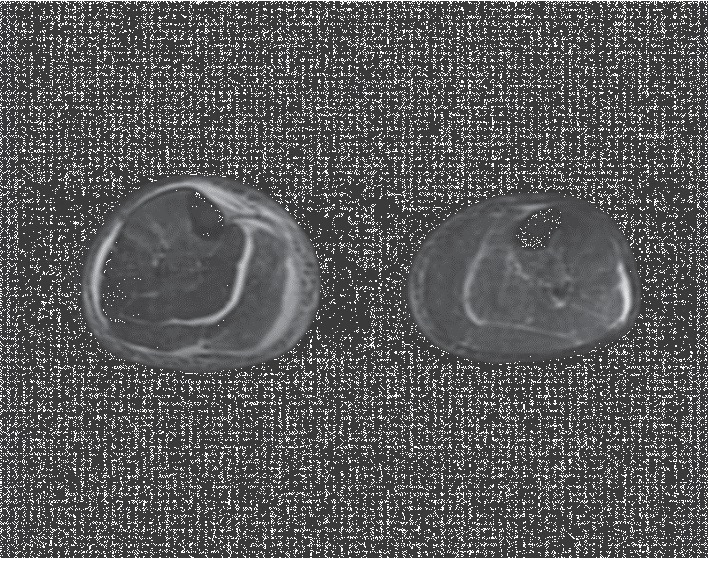
Horizontal section of magnetic resonance imaging (MRI) of lower extremities, with and without contrast, showing edema within the fascial compartments between the right gastrocnemius and right soleus muscles, as well as in the superficial fascia. A crescent-shaped subfascial fluid collection was also noted over the medial head of the right gastrocnemius muscle, indicative of fasciitis.

On the first day of admission, a right lower extremity ultrasound was ordered due to the pain and swelling in his right calf. This study was negative for deep vein thrombosis. The following day, a magnetic resonance imaging (MRI) of the right lower leg, with and without contrast (Figure 3), showed edema within the fascial compartments between the gastrocnemius and soleus muscles, as well as in the superficial fascia. A crescent-shaped subfascial fluid collection was also noted over the medial head of the gastrocnemius muscle, indicative of right lower leg fasciitis. The decision was made to proceed with incision and drainage of the fluid collection, in addition to a debridement of the right leg necrotizing fasciitis. Abscess drainage resulted in approximately 500 ml of purulent foul-smelling fluid, some of which was sent for gram stain and culture. A wash out of the right lower extremity wounds would be performed several days later with subsequent placement of negative pressure wound dressing.

Infectious disease was consulted on the second day of admission. At this time, the patient was being treated with IV cancomycin and piperacillin/tazobactam. The decision was made to add clindamycin to help with possible toxin production and doxycycline due to concern for Brucella and Coxiella. Initial blood cultures had been negative; however, gram-negative anaerobic bacteria were isolated from the fluid sample taken off the right leg abscess. This was later identified to be *Fusobacterium necrophorum*. On the seventh day of admission, antibiotics were deescalated to meropenem based on susceptibilities. The patient had improved clinically since his admission, but he continued to spike fevers. A follow-up chest CT showed persistence of numerous lung abscesses in the subpleural area with risk of bronchopleural fistula evolution. Due to concern over persistent fevers and empyema, despite multiple chest tube placements, the patient was transferred to a tertiary care center for further evaluation.

The patient remained in the ICU at the tertiary care center for another week where he had two more surgeries on his right leg. He also underwent a lung decortication surgery. The patient reported that in his mind, the lung decortication was a turning point in his recovery. He was transferred to a step-down unit and then discharged to home on a four-week course of oral clindamycin. He also underwent outpatient physical therapy. He has since made a complete recovery and was able to continue his undergraduate education.

## 3. Discussion

The increased use of antibiotics to treat pharyngitis has led to a decreased occurrence of Lemierre's Syndrome, earning it the moniker “the forgotten disease.” One source states incidence in the early 1990s was as low as 0.8–1.5 per million persons per year [8]. However, there have been an increased number of cases reported over the last 20 years [9]. There have been several proposed reasons for this increase in incidence. One possible cause is a decreased use of antibiotics to treat uncomplicated pharyngitis. Routine use of antibiotics to treat pharyngitis has been discouraged over recent years, which could be contributing to the progression of *Fusobacterium* infection into Lemierre's syndrome. Another postulate is that improved anaerobic culturing techniques have led to increased detection of *Fusobacterium* infection. In this case, the increased number of cases would be due to more sensitive testing rather than an actual increase in incidence. Yet another theory is that the increased number of cases is due to emerging antibiotic resistance amongst *Fusobacterium* species. A recent study showed the resistance rate to erythromycin was as high as 15% amongst strains of *Fusobacterium necrophorum* [10]. Penicillin resistance has also been reported due to production of beta-lactamase by some strains of *Fusobacterium necrophorum* [11]. Appropriate antibiotic selection is discussed later in the section.

Regardless of the cause of the increasing incidence, a high level of clinical suspicion is required to make the diagnosis of Lemierre's syndrome. The disease is often initially misdiagnosed as a viral pharyngitis, especially mononucleosis [2]. Several clinical clues can be helpful in distinguishing Lemierre's syndrome from mononucleosis. For instance, mononucleosis typically presents with more generalized lymphadenopathy in contrast to the more localized cervical lymphadenopathy of Lemierre's syndrome. Another clue, and one of the most typical findings of a Lemierre's syndrome, is the presence of lung infiltrates. In young patients with pharyngitis, the presence of concurrent lung lesions should raise the clinical suspicion for Lemierre's syndrome. Critical to the diagnosis of Lemierre's syndrome is the prompt collection of blood cultures and the culturing of any fluid drained from abscesses that may be present. However, *Fusobacterium necrophorum* takes at least 48 hours to grow in culture and sometimes as long as seven days [2], so clinicians may find it necessary to treat empirically while awaiting culture results. There are also multiple case reports of atypical presentations of Lemierre's syndrome, including with ARDS and septic shock [12], further adding to the diagnostic challenge that the disease presents.

Once Lemierre's syndrome is suspected, administration of IV antibiotics should begin immediately. However, the optimal antibiotic regimen is not agreed upon. Most reports have recommended use of high-dose beta-lactamase-resistant beta-lactam antibiotic in conjunction with metrondizole. However, use of clindamycin alone has also been shown to be effective [7]. Of note is the slow response time of Lemierre's syndrome to antibiotics. Time from antibiotic administration to resolution of fever can last upwards of 8–12 days [2]. The slow response time is thought to result from sequestration of the infection with resulting poor penetration of antibiotics. Another point of controversy is whether to administer anticoagulants in treatment of Lemierre's syndrome due to the propensity for thrombus formation. Some reports have supported use of anticoagulants in addition to systemic antibiotics, but no clear guidelines exist [13]. A retrospective study has shown that use of anticoagulants did not affect thrombus outcome in cases of Lemierre's syndrome, with thrombus resolution occurring in all 24 cases regardless of anticoagulant use [14]. However, anticoagulants may be indicated for high-risk patients, such as those with extensive internal jugular thrombosis, or those who have not responded to antimicrobial therapy [13].

While Lemierre's syndrome remains a rare condition, it nonetheless is associated with considerable morbidity and mortality. With an increased awareness of the condition, physicians will be more likely to consider it in a differential diagnosis. Prompt recognition by the treating physician and the timely administration of systemic antibiotics are key to successful outcomes. However, the use of anticoagulants remains controversial. Physicians would benefit from using a case-by-case approach when making a decision regarding the use of anticoagulation. These patients are also more likely to develop complications secondary to septic embolic phenomenon. Transfer to a larger hospital center with greater surgical capabilities may be a prudent decision.

## Additional Points

*Learning Points*. (1) Lemierre's syndrome is a relatively rare disease process that results from oropharyngeal infection involving *Fusobacterium necrophorum*; however, increasing prevalence has been reported. Local invasion into the internal jugular vein with metastatic spread of septic microemboli is the typical progression of illness. (2) The diagnosis should be considered in young, otherwise healthy patients who present with pharyngitis and lung abscess concurrently. However, it can also present more atypically, as with the lower extremity necrotizing fasciitis seen in our patient. (3) Treatment is centered around early antibiotic administration and infection source control. Antimicrobial resistance to erythromycin and penicillin has been documented and should be taken into consideration when selecting initial antibiotics.
